# Enlargement of the Liver in Diphtheria

**Published:** 1905-09-16

**Authors:** 


### ENLARGEMENT OF THE LIVER IN DIPHTHERIA.

Hepatic enlargement in diphtheria has been noticed chiefly in connection with the morbid anatomy of the disease. Dr. Rolleston,[1] however, believes that it is a sign of some clinical and prognostic importance, and that more attention should be directed to it at the bedside. He studied 310 cases of diphtheria with special reference to enlargement of the liver, and he found appreciable increase in size of this organ in 36 cases, or 11·6 per cent. All of these cases were classed as "very severe," or "severe," and the sign was observed in 70·5 of all cases which were considered severe. The prognostic significance of hepatomegaly is indicated by the fact that the mortality among the cases in which the liver was enlarged was 44·4 per cent., while of the total 310 cases only 7·7 per cent. ended fatally.

The two principal complications of diphtheria, albuminuria and paralysis, were more frequent in those cases in which enlargement of the liver occurred than in other cases. Thus albuminuria was present in all the thirty-six instances of hepatomegaly and in fifteen the albumen was abundant and persistent, and 75 per cent. of the survivors suffered from post-diphtheritic paralysis.

The actual enlargement is due, Dr. Rolleston says, to vascular engorgement associated with the failing heart and, to a lesser extent, to fatty changes in the liver cells. The onset of hepatomegaly is most frequent between the fifth and eleventh days of the disease. The sign may be accepted as an alarm signal by its occasionally preceding the physical signs of cardiac paralysis. It also serves to confirm the gravity of other symptoms, which, taken by themselves, are of less value. This particularly applies to cardiac vomiting, which not unfrequently precedes the physical signs denoting its true nature. Enlargement of the liver, even with an apparently normal heart, when associated with vomiting is, therefore, of very grave prognosis, and is often a prelude to fatal cardiac paralysis. In conclusion, Dr. Rolleston says that determination of the size of the liver should form as essential a part in the routine examination of the patient in all severe cases of diphtheria during the first three weeks of the disease as the investigation of the condition of the heart and pulse.

[1] Metropolitan Asylums Board's Annual Report, 1904.

